# Lower Glucose Effectiveness Is Associated with Postprandial Hyperglycemia in Obese/Overweight Men, Independently of Insulin Secretion

**DOI:** 10.3390/metabo12111022

**Published:** 2022-10-25

**Authors:** Ichiro Kishimoto, Akio Ohashi

**Affiliations:** 1 Department of Endocrinology and Diabetes, Toyooka Public Hospital, 1094 Tobera Toyooka, Hyogo, Toyooka 668-8501, Japan; 2 Environment, and Total Quality Management Division, NEC Corporation, 5-7-1 Minato-ku, Shiba, Tokyo 108-8001, Japan

**Keywords:** glucose effectiveness, postprandial hyperglycemia, obesity, walking, noodle ingestion

## Abstract

The role of glucose effectiveness on postprandial hyperglycemia in daily life is not fully studied. Here, we examined the association between SgIo, an index of glucose effectiveness calculated from a 75 g oral glucose tolerance test, and the indices of hyperglycemia in obese/overweight men. SgIo was significantly associated with 1,5-anhydroglycitol, a biochemical marker for postprandial hyperglycemia. The receiver operating characteristic analyses of SgIo and oral disposition index for detecting the subjects with 1,5-anhydroglycitol < 14 μg/mL revealed that the areas under the curves were 0.77 and 0.76, while the cutoff points (sensitivity, selectivity) were 2.53 (0.9, 0.7) and 2.06 (0.36, 0.79), respectively. Both the SgIo < 2.53 category and the disposition index < 2.06 category were significantly associated with the percentages of meals with postprandial glucose levels ≥ 200 mg/dL, and the percentages of time when continuous glucose monitoring sensor readings were ≥200 mg/dL. After adjustment with disposition index, 45.5% of the subjects with the SgIo < 2.53 category had their 1,5-anhydroglycitol < 14 μg/mL, while, in the SgIo ≥ 2.53 category, 3.6% of the subjects had the hyperglycemia (*p* < 0.001). In addition, there were tendencies toward higher and lower SgIo quartile categories in subjects with walking (≥8000 steps) ≥60% of days and with noodle ingestion ≥20% of meals, respectively (*p* for trend, 0.008 and 0.038). In conclusion, lower glucose effectiveness is associated with postprandial hyperglycemia in the daily life of obese/overweight men, independently of insulin secretion. Lifestyles such as habits of walking and noodle ingestion are significantly associated with higher and lower glucose effectiveness, respectively.

## 1. Introduction

Glucose disposal is mediated by both insulin-dependent and insulin-independent mechanisms. While the former is determined by the interaction of insulin sensitivity and insulin secretion, the latter, termed glucose effectiveness (Sg), is the ability of glucose itself to increase peripheral glucose utilization and inhibit hepatic glucose production, independently of the insulin action [1]. Sg is a major determinant of intravenous glucose tolerance [2] and has been regarded as an important mechanism for maintaining normoglycemia [3]. It is known that glucose effectiveness is reduced in type 2 diabetes [3] and obesity [4,5].

Nonetheless, mechanisms that regulate Sg are poorly understood and data on its impact on glucose intolerance is only limited. This is because to estimate Sg, either a euglycemic clamp or frequently sampled intravenous glucose tolerance test [3], both of which are invasive, laborious, and time-consuming, are necessary. To overcome the issue, Nagasaka et al. developed an index of Sg derived from a standard 75 g oral glucose tolerance test (OGTT) and named it the oral Sg index (SgIo), which accurately predicted Sg [6].

Recently, we have shown that, in middle-aged men with overweight/obesity, a substantial proportion exhibited elevated glucose levels above the recommended target for diabetes management (postprandial hyperglycemia) during continuous glucose monitoring (CGM) [7] and that lifestyles such as snacking and physical inactivity serve as the major drivers of the postprandial hyperglycemia independently of β-cell function [8]. In the present study, to determine the role of Sg on postprandial hyperglycemia in obese/overweight, the associations between SgIo and 1,5-anhydroglycitol (AG), a clinical index of postprandial hyperglycemia, were examined with the adjustment of disposition index, a measure for insulin secretion relative to insulin sensitivity. In addition, the relationships between SgIo and postprandial hyperglycemia determined by blood glucose self-monitoring (BGM) or CGM were investigated. Furthermore, the associations between SgIo and lifestyle behaviors such as diets and exercise, the two major modifiable factors for hyperglycemia, were assessed.

## 2. Materials and Methods

Study protocol: A total of 50 obese/overweight (body mass index, BMI ≥ 25 kg/m^2^), middle-aged (age 50–65 years) male participants were recruited. On the first experimental day, the venous blood was drawn from the cubital vein and the fasting plasma glucose (FPG), serum glycated hemoglobin (HbA1c), and plasma 1,5-AG, the levels of which decrease during times of hyperglycemia above 180 mg/dL [9,10,11], were measured using standard laboratory procedures after overnight fasting (≥12 h). Glucose, HbA1c, and 1,5-AG were determined using the enzymatic hexokinase, high-performance liquid chromatography (HPLC), and enzymatic colorimetric methods, respectively. A 2 h, 75 g OGTT was then performed. The cutoff 2 h plasma glucose levels of 140 mg/dL and 200 mg/dL were used to diagnose normal (NGT), impaired glucose tolerance (IGT), and diabetes mellitus (DM). After OGTT, subjects were asked to create a seven-point BGM profile (preprandial, 1~2 h postprandial, and pre-bedtime) by using a glucometer (Glutest Neo Alpha; Sanwa Kagaku Kenkyusho Co., Osaka, Japan). In addition, participants are instructed to wear CGM devices (iPro™2 Professional CGM, Medtronic, MN, USA) during the study period and to calibrate the sensor according to the manufacturer’s specifications four times throughout the day. The experiment was performed from the morning of the first day to the morning of the 7th day.

Sg-related index: SgIo, an index of Sg determined from 75 g OGTT data, was calculated using Nagasaka’s equation [6]. In brief, SgIo (mg/dL/min) = (a − b * c)/120.

where:

(a) (PPG without insulin and Sg) The post-load glucose (PPG) without the action of insulin and glucose, which was calculated as: fasting plasma glucose (mg/dL) + (0.75 * 75,000)/(0.19 * body weight (kg) * 10).
(b) (PPG without insulin/with Sg) The value calculated based on the relationship between the whole-body insulin action quantified by oral disposition index (DIo) and 2hPG, the glucose level at 2 h after 75 g oral glucose challenge, across the spectrum of glucose tolerance. Here, (the mean minus 3SD, standard deviation) of log10[DIo] (−1.158) was substituted for DIo equaling 152, 213, and 342 mg/dL for NGT, IGT, and DM, respectively.
(c) (adjustment factor) The ratio of 2hPG/2hPGE constitutes the required adjustment factor, where 2hPGE is the expected 2hPG, which was obtained from the regression between DIo and 2hPG [6].

Insulin-related indices: Indirect indices of insulin secretion or insulin resistance were determined based on simultaneous measurements of blood glucose and insulin concentrations (IRI, immunoreactive insulin) under fasting conditions or during the OGTT. The homeostatic model assessment (HOMA)-β was determined by the following formulae: fasting insulin × 360/(fasting glucose − 63). HOMA-R, the insulinogenic index, the Matsuda index, and DIo (the product of the insulinogenic index and Matsuda index) were calculated online at http://mmatsuda.diabetes-smc.jp/MIndex.html (accessed on 30 August 2022). Formulae for HOMA-R, insulinogenic index, and Matsuda index were, (OGTT PG 0 * OGTT IRI 0)/405, (OGTT IRI 30 − OGTT IRI 0)/(OGTT PG 30 − OGTT PG 0), 10000/SQRT((OGTT PG 0 * OGTT IRI 0) * ((OGTT PG 0 + OGTT PG 30 * 2 + OGTT PG 60 * 3 + OGTT PG 120 * 2)/8 * (OGTT IRI 0 + OGTT IRI 30 * 2 + OGTT IRI 60 * 3 + OGTT IRI 120 * 2)/8)), respectively. 

Lifestyle-related indices: During the study period, participants ate, drank, and moved at their own discretion. They were asked to keep daily logs of food intake and exercise, and to take photographs of every meal/drink content with a date and time stamp with a digital camera (COOLPIX, Nikon, Tokyo, Japan). The percentage of occasions on which the participant consumed food items per meal was calculated. For the analyses including the frequency of noodle ingestion, participants who did not complete the recording of the diet logs during the study were excluded (*n* = 8). In the present study, daily walking steps during the study were assessed using smartwatch-type activity trackers (PULSENSE, EPSON, Tokyo, Japan). For the analyses including walking steps, participants who did not complete the recording of the steps during the study were excluded (*n* = 1). Since walking at least 7000–10,000 steps a day reduced middle-aged people’s risk of premature death [12], we defined the “walking day” as the day when subjects walked ≥8000 steps. 

Data analysis for CGM: As indicators of postprandial hyperglycemia, percentages of postprandial peaks above the selected glucose thresholds (i.e., 140, 180, and 200 mg/dL), were calculated using all CGM glucose data obtained during the study. The glucose concentrations corresponding to the proposed cutoff points as clinical targets were used as the thresholds. For the analysis including CGM data, participants with CGM readings <720 (60 h) were excluded (*n* = 7).

Statistical analysis: Baseline data are expressed as the median and interquartile range (IQR) for all participants or stratified by the SgIo category. Subjects with BGM recordings <20 were excluded (*n* = 5) from the analyses including BGM. For the contingency table, Fisher’s exact test was used to calculate the significance of the deviation from a null hypothesis. To measure the strength and direction of association between anthropometric or biochemical parameters and SgIo, Spearman’s rank-order correlation coefficients (ρ) were calculated. The Mann–Whitney *U* test was used for the comparison of continuous variables based on the categorical data. Participants were categorized by SgIo (<2.53 or ≥2.53), DIo (<2.06 or ≥2.06), frequency of walking ≥8000 steps a day (<60% or ≥60% of days), and frequency of noodle ingestion (<20% or ≥20% of meals). The cutoff values for SgIo and DIo have obtained from the receiver–operating characteristic (ROC) curves for detecting the 1,5-AG < 14 μg/mL categories, while that for the frequency of noodle ingestion was calculated from the ROC curve for detecting the SgIo < 2.53 category.

The Cochran–Armitage trend test was designed to assess the null hypothesis that there are no ordered differences in the proportions of the SgIo quartile categories across the walking day category or of the frequency of noodle ingestion category.

Multiple linear regression was fitted for 1,5-AG with SgIo and DIo as the predictive factors. The normality of residuals was validated by the Shapiro–Wilk test (W = 0.987, *p* = 0.854). The variance inflation factor calculated for each predictor was 1.03, indicating that multicollinearity could be safely ignored. In addition, a multiple logistic regression model was constructed to examine the association between the 1,5-AG < 14 μg/mL category and independent variables including the SgIo and DIo categories. To integrate a two-level categorical variable into the regression models, a dummy variable with two values was created by assigning 1 for the objective category and −1 for the control category. The odds ratios of having the 1,5-AG < 14 μg/mL categories were calculated via the maximum likelihood method in the logistic regression models. 

The ROC curve analysis was applied to measure the diagnostic accuracy of SgIo and DIo for predicting the 1,5-AG < 14 μg/mL categories. For a measure of goodness of fit for binary outcomes in a logistic regression model, the area under the curve (AUC) was computed. The cutoff point was determined via the Youden index to maximize the overall accuracy of the classification rate and assign equal weight to the sensitivity and the specificity.

Statistical significance was defined as a *p*-value of <0.05. 

Ethics: The study was performed in accordance with the principles established by the Helsinki Declaration and approved by the institutional review board of Toyooka Public Hospital (#146; 3 October 2017) and the Japan Conference of Clinical Research review board (JCCR#3-132; 21 October 2016). Written informed consent was obtained from all subjects prior to study enrollment.

## 3. Results

### 3.1. Baseline Characteristics

The characteristics of the study subjects were shown in Table 1. The median (IQR) age and BMI were 54 (52–58.3) and 27.8 (26.7–29.2), respectively. The median (IQR) HbA1c and 1,5-AG levels were 36 (33–38) mmol/mol and 19.7 (14.9–24.0) µg/mL. In 44% of subjects, HOMA-R was ≥2.0. In 18%, insulinogenic index was <0.4. Median (IQR) Matsuda index, DIo, and SgIo were 4.06 (2.82–6.39), 3.14 (1.91–5.69), and 2.59 (2.2–2.87), respectively. The 2 h 75 g OGTT revealed that 74%, 24%, and 2% of participants meet the criteria for NGT, IGT, and DM, respectively. Medians (IQR) at 1 h and 2 h post-challenge glucose levels during OGTT were 168 (132.8–194) mg/dL and 110.5 (93.5–141.5) mg/dL, respectively. During the study, the median (IQR) number of self-monitored blood glucose measurements obtained per person was 45 (36.8–47). During the same period, a total of 1959 (1940–1973) CGM sensor glucose data was recorded, which showed approximately 60% of postprandial glucose peaks exceed 140 mg/dL. Medians (IQR) of maximal BGM and CGM glucose levels were 187 (169.5–201.5) and 187 (169–216) mg/dL, respectively. The median daily walking steps were 7478 (5051–9408). A quarter of subjects walk ≥8000 steps/day in ≥60% of days (approximately 4 days a week). In 15 (10–25)% of meals, subjects eat noodles during the study period.

### 3.2. Association between SgIo and Biochemical Indices

As shown in Table 2, SgIo is significantly correlated with HbA1c and 1,5-AG. In addition, lower SgIo was associated with higher HOMA-R. The median (IQR) SgIo is 2.7 (2.39–2.93) in the HOMA-R < 2.5 category (*n* = 39), while that is 2.19 (1.74–2.58) in the HOMA-R ≥ 2.5 category (*n* = 11) (*p* = 0.005).

### 3.3. 1,5-AG and Postprandial Hyperglycemia

For the index of postprandial hyperglycemia, 1,5-AG was chosen in the present study. The levels of 1,5-AG were significantly associated with CGM indices for hyperglycemia (Table 3). For categorical analysis of 1,5-AG, 14 μg/mL, the reference value in Japan, was used for the cutoff. In the present study, 33% of subjects with 1,5-AG < 14 μg/mL had postprandial CGM glucose ≥180 mg/dL peaks in more than 40% of meals, while 2.9% of subjects with 1,5-AG ≥ 14 μg/mL did.

### 3.4. SgIo and Postprandial Hyperglycemia

The ROC analysis revealed that SgIo significantly predicts the 1,5-AG category (*p* = 0.02). The AUC and the cutoff point (sensitivity, selectivity) of SgIo for detecting the 1,5-AG < 14 μg/mL category are 0.77 and 2.53 (0.9, 0.7), respectively. As shown in Table 4, the SgIo < 2.53 category was significantly associated with indices for postprandial hyperglycemia (180–200 mg/dL) detected by BGM or CGM.

### 3.5. DIo and Postprandial Hyperglycemia

DIo also significantly predicts the 1,5-AG category (*p* = 0.011). The AUC of the ROC curve and the cutoff point (sensitivity, selectivity) of DIo for detecting 1,5-AG < 14 μg/mL are 0.76 and 2.06 (0.36, 0.79), respectively. As shown in Table 5, the DIo < 2.06 category was significantly associated with the percentage of postprandial BGM ≥ 200 mg/dL, percent of time CGM sensor reading ≥180 mg/dL, and ≥200 mg/dL.

### 3.6. Insulin-Independent Association of SgIo and Postprandial Hyperglycemia 

Multi-variate analysis showed that lower SgIo and lower DIo were independently associated with 1,5-AG or with the 1,5-AG < 14 μg/mL category. After adjustment of DIo, the odds of the SgIo < 2.53 category for having the 1,5-AG < 14 μg/mL category was 20.8 (95% confidence interval, 3.2–413.1) times higher than that of the SgIo ≥ 2.53 category (*p* < 0.001). In the SgIo < 2.53 category, 45.5% of the subjects had their 1,5-AG less than 14 μg/mL, while, in the SgIo ≥ 2.53 category, 3.6% of the subjects had (*p* < 0.001). 

On the other hand, after adjustment of SgIo, the odds of the DIo < 2.06 category for having the 1,5-AG < 14 μg/mL category was 5.1 (95% confidence interval, 1.1–25.6) times higher than that of the DIo ≥ 2.06 category (*p* = 0.033). In the DIo < 2.06 category, 42.9% of the subjects had their 1,5-AG less than 14 μg/mL, while, in the DIo ≥ 2.06 category, 13.9% of the subjects had (*p* = 0.026).

When the proportions of the 1,5-AG < 14 μg/mL category in the SgIo < and ≥2.53 categories were examined with stratification by the DIo category, the proportion in the lower SgIo category is significantly higher than those in the upper SgIo category, irrespectively of the DIo category (Table 6). On the other hand, when the proportions of the 1,5-AG < 14 μg/mL category in the DIo < and ≥2.06 categories were examined with stratification by the SgIo category, in subjects with the lower SgIo category, the lower DIo category was associated with the postprandial hyperglycemia twice as much as the higher DIo category (66.7% vs. 30.8%). Nonetheless, the difference between the lower and upper DIo categories did not reach a statistical significance in either SgIo category as shown in Table 6.

### 3.7. SgIo and Lifestyles

The associations between SgIo and lifestyle-related factors were next examined. Here, the walking days were defined as the day when subjects walk ≥8000 steps/day, and subjects were divided by if their walking days are ≥60% of the total study period (i.e., approximately 4 days per week). In subjects with their walking days < 60% (*n* = 37), median (IQR) SgIo was 2.53 (2.08–2.75), while in those with their walking days ≥ 60% (*n* = 12), that was 2.88 (2.49–2.98) (*p* = 0.032). 

The ROC of the frequency of noodle ingestion for detecting the SgIo < 2.53 category showed the AUC of 0.76 and the optimal cutoff value (sensitivity, selectivity) of 20% of meals (0.67, 0.75) (*p* = 0.015). In subjects with their frequency of noodle ingestion < 20% (*n* = 27), median (IQR) SgIo was 2.68 (2.41–2.96), while in those with frequency of noodle ingestion ≥20% (*n* = 15), that was 2.37 (1.74–2.86) (*p* = 0.048).

Proportions of the SgIo quartile category stratified with lifestyle-related categories were next examined. As shown in Table 7, compared to subjects with walking days < 60% (approximately 4 days per week), subjects with walking days ≥ 60% showed a tendency toward higher SgIo quartile categories (*p* for trend = 0.008). On the contrary, compared to subjects with a frequency of noodle ingestion < 20%, subjects with a frequency of noodle ingestion ≥ 20% (approximately once every other day) showed a tendency toward lower SgIo quartile categories (*p* for trend = 0.038).

## 4. Discussion

In the present study, lower SgIo, an index of Sg, is associated with higher indices of postprandial hyperglycemia in obese/overweight men. The association was independent of DIo, a maker of insulin secretory capacity relative to insulin resistance. In addition, SgIo is lower in subjects with a lower frequency (<60% of days) of walking ≥8000 steps a day, or with a higher frequency of noodle ingestion (≥20% of meals).

In normal individuals, it is reported that approximately half of the glucose disposal during OGTT is due to Sg; whereas, in the insulin-resistant obese individual, 83% of glucose disposal occurs independently of the dynamic insulin response [13]. Reduced Sg is a major contributor to obesity-associated glucose intolerance as well as a strong risk factor for the development of type 2 diabetes [14,15]. In the present study, 88.2% of total glucose disposal is attributed to Sg in obese/overweight men, suggesting that Sg is a key determinant of glucose intolerance, and the defect of Sg could lead to post-load hyperglycemia. In fact, lower SgIo is associated with indices for postprandial hyperglycemia determined by 1,5-AG, BGM, and CGM (Table 2 and Table 4).

Since previous studies showing a reduction in glucose effectiveness in type 2 diabetes or obesity were based on experimental methods such as the glucose clamp or the frequently sampled intravenous glucose tolerance test, it is not feasible to extrapolate the results into public health or daily clinical settings. In addition, no study examined the association between glucose effectiveness and postprandial hyperglycemia or lifestyle factors in daily life. Since, without continuous glucose monitoring, postprandial hyperglycemia cannot be precisely determined, it is not easy to evaluate postprandial hyperglycemia of subjects without diabetes in daily life. The present study revealed that, with the utilization of the OGTT-based index, it is possible to select persons prone to postprandial hyperglycemia in their daily lives. Since early recognition of postprandial hyperglycemia is a key to preventing the development and worsening of type 2 diabetes, the findings presented in our study are of importance for public health and of clinical significance.

It is reported that Sg and disposition index independently predict conversion to diabetes across various stages of glucose tolerance and obesity [15]. However, it has not been known the roles of Sg and DIo on postprandial hyperglycemia in daily life. In the present study, only 3.6% of subjects with the higher SgIo category develop postprandial hyperglycemia (1,5-AG < 14 μg/mL), while 45.5% of subjects with the lower SgIo category do. In addition, the effect of the Sg category (< or ≥2.53) on postprandial hyperglycemia was observed irrespectively of the DIo category (< or ≥2.06) (Table 6). Furthermore, a multiple logistic regression model showed that the effect of SgIo on the 1,5-AG < 14 μg/mL category was independent of DIo. These results suggest that, in obese/overweight men, Sg plays a key role in the development of postprandial hyperglycemia in daily life independently of insulin secretion. 

Exercise training is known to confer significant improvements in Sg [16,17,18,19]. It is suggested that exercise induces GLUT4 translocation to the plasma membrane [20] and increases AMP-activated protein kinase (AMPK) [21] which leads to an insulin-independent increase in glucose transport following exercise. However, the effect of light daily exercise such as walking on Sg has not been fully studied. In the present study, subjects with walking habits (≥8000 daily steps, ≥60% of days) had higher SgIo than those without (median, 2.88 (IQR 2.49–2.98) vs. 2.53 (2.08–2.75), *p* = 0.032). In addition, the higher SgIo quartile category is significantly associated with subjects with walking habits (Table 7). Half of the subjects with walking habits belong to the highest SgIo quartile category, while 16.2% of those without the habits do. It is, therefore, suggested that daily exercise habits are vital to maintaining high Sg. 

In the present study, subjects with frequent noodle ingestion are associated with lower SgIo categories. As shown in Table 7, 40% of subjects with noodle ingestion habits (≥20% of meals) belong to the lowest SgIo quartile category, while 18.5% of those without the habits do. On most occasions, study subjects ate instant noodles, in which the average content of saturated fatty acids is considerably high among cereal foods [22]. Since high-fat diets reduce the contribution of Sg to glucose disposal to approximately 40% [23], the reduced SgIo in subjects with noodle ingestion habits may be explained by an excess of high-fat intake. As shown in Table 2, low SgIo is associated with higher fasting insulin levels and HOMA-R, suggesting reduced Sg in the presence of hepatic insulin resistance. Since higher noodle consumption is associated with higher HOMA-R [24] and since hepatic insulin resistance is often accompanied by fatty liver, lower SgIo in the frequent noodle ingestion group could be attributable at least partly to hepatic steatosis. 

Limitations of the study include: (1) the oral surrogate of Sg (i.e., SgIo) was used instead of Sg determined by either euglycemic clamp or frequently sampled intravenous glucose tolerance test; (2) a small sample size of obese/overweight men could make it hard to extrapolate the finding to the general population; (3) it is not possible to delineate the components of Sg, i.e., a decrease in endogenous hepatic glucose production and/or an increase in whole-body glucose uptake; (4) for the estimate of the disposition index, DIo (the Matsuda Index × insulinogenic index) is adopted in the present study, which includes post-load insulin sensitivity in its formulae. While another estimate of the oral disposition index, insulinogenic index × 1/fasting insulin [25], is also widely used, the values of two DIos were similar in the present study (R^2^ = 0.96); (5) although indices other than the Matsuda index are also proposed for estimation of insulin sensitivity [26,27], we do not have data regarding plasma glucose and serum insulin levels at 90 min after 75 g glucose load, which are necessary for the calculation of the indices; and (6) in the present study, we focused not on food composition but on food items. Therefore, if other starch-rich food also had similar effects as noodles is not known.

In conclusion, lower Sg is associated with postprandial hyperglycemia in obese/overweight men, independently of insulin secretion. Lifestyles such as walking habits (≥8000 daily steps, ≥60% of days) or avoiding frequent pre-cooked noodle ingestion (≥20% of meals) might lead to higher Sg and prevent postprandial hyperglycemia.

## Figures and Tables

**Table 1:** The median and interquartile range of the anthropometric, lifestyle, or biochemical indices.

Parameter	Median	IQR	*n*
Age, years	54	52–58.3	50
BMI, kg/m^2^	27.8	26.6–29.2	50
HbA1c, mmol/mol	36	33–38	50
1,5-AG, μg/mL	19.7	14.9–24.0	50
HOMA-R	1.86	1.13–2.40	50
Insulinogenic Index	0.75	0.51–1.34	50
Matsuda index	4.06	2.82–6.39	50
DIo	3.14	1.91–5.69	50
SgIo, mg/dL/min	2.59	2.20–2.87	50
			
OGTT PG 0 min, mg/dL	91.5	85–97	50
OGTT PG 30 min, mg/dL	151	134.3–175.3	50
OGTT PG 60 min, mg/dL	168	132.8–194	50
OGTT PG 120 min, mg/dL	110.5	93.5–141.5	50
OGTT IRI 0 min, μU/mL	7.9	5.2–10.4	50
OGTT IRI 30 min, μU/mL	54.3	33.2–75.2	50
OGTT IRI 60 min, μU/mL	66.3	47.7–130.3	50
OGTT IRI 120 min, μU/mL	51.8	31.4–96.4	50
			
BGM total, counts	45	36.8–47	48
BGM post, counts	17	13–19	48
BGM min, mg/dL	70.5	62.3–77.8	48
BGM max, mg/dL	187	169.5–201.5	48
BGM mean, mg/dL	115.5	107–123	48
BGM SD, mg/dL	26.1	21.9–30.2	48
BGM CV, %	23.0	19.5–25.4	48
			
CGM total, counts	1959	1940–1973	43
CGM min, mg/dL	67	53–76	43
CGM max, mg/dL	187	169–216	43
CGM mean, mg/dL	113	104–119	43
CGM SD, mg/dL	20.8	16.7–23.4	43
CGM CV, %	18.3	15.5–20.7	43
% of ≥200 peaks ^†^	0	0–6.7	43
% of ≥180 peaks ^†^	6.7	0–21.7	43
% of ≥140 peaks ^†^	60	25–78.3	43
			
Steps mean, steps/day	7478	5051–9408	48
≥8000 steps, % of days	28.6	14.3–60	48
≥10,000 steps, % of days	14.3	14.3–39.3	48
<3000 steps, % of days	0	0–28.6	48
			
Noodle, % of meals	15	10–25	42
Curry, % of meals	0	0–6	42
Sushi, % of meals	0	0–5	42
Donburi, % of meals	5	0–12	42

BMI, body mass index; 1,5-AG, 1,5-anhydroglucitol; HOMA, homeostatic model assessment; DIo, oral disposition index; SgIo, glucose effectiveness determined from 75 g oral glucose tolerance test data; OGTT, oral glucose tolerance test; PG, plasma glucose level; IRI, serum immunoreactive insulin level; BGM, blood glucose self-monitoring; SD standard deviation; CV, coefficient of variation; CGM, continuous glucose monitoring; IQR, interquartile range. ^†^, The percentages of meals with postprandial CGM peaks above the indicated thresholds.

**Table 2:** Spearman’s rank-order correlation coefficients (ρ) of associations between SgIo, and anthropometric or biochemical indices.

Parameter	ρ	*p*
Age, years	−0.09	0.538
BMI, kg/m^2^	−0.47	0.001 *
HbA1c, mmol/mol	−0.37	0.007 *
1,5-AG, μg/mL	0.37	0.009 *
HOMA-R	−0.42	0.002 *
Insulinogenic index	−0.08	0.593
Matsuda index	0.45	0.001 *
DIo	0.20	0.158
		
OGTT PG 0 min, mg/dL	0.02	0.889
OGTT PG 30 min, mg/dL	−0.14	0.325
OGTT PG 60 min, mg/dL	−0.19	0.194
OGTT PG 120 min, mg/dL	−0.68	0.001 *
OGTT IRI 0 min, μU/mL	−0.42	0.003 *
OGTT IRI 30 min, μU/mL	−0.22	0.116
OGTT IRI 60 min, μU/mL	−0.13	0.357
OGTT IRI 120 min, μU/mL	−0.53	0.001 *

SgIo, glucose effectiveness determined from 75 g oral glucose tolerance test data; BMI, body mass index; 1,5-AG, 1,5-anhydroglucitol; HOMA, homeostatic model assessment; DIo, oral disposition index; OGTT, oral glucose tolerance test; PG, plasma glucose level; IRI, serum immunoreactive insulin level; * *p* < 0.05.

**Table 3:** Spearman’s rank-order correlation coefficients (ρ) of associations between indices for postprandial hyperglycemia and 1,5-AG.

Parameter	ρ	*p*
Age, years	−0.18	0.259
BMI, kg/m^2^	0.1	0.506
HbA1c, mmol/mol	−0.24	0.128
Insulinogenic index	0.26	0.097
Matsuda index	0.13	0.41
DIo	0.28	0.072
CGM max, mg/dL	−0.34	0.002 *
time CGM ≥ 200, % ^†^	−0.34	0.028 *
time CGM ≥ 180, % ^†^	−0.38	0.013 *
time CGM ≥ 140, % ^†^	−0.21	0.176
% of ≥200 peaks ^‡^	−0.31	0.043 *
% of ≥180 peaks ^‡^	−0.32	0.035 *
% of ≥140 peaks ^‡^	−0.05	0.73

1,5-AG, 1,5-anhydroglucitol; BMI, body mass index; DIo, oral disposition index; CGM, continuous glucose monitoring. ^†^, The percentages of time when CGM sensor readings were above the indicated thresholds (i.e., 200, 180, and 140 mg/dL); ^‡^, The percentages of meals with postprandial CGM peaks above the indicated thresholds (i.e., 200, 180, and 140 mg/dL); * *p* < 0.05.

**Table 4:** The median and interquartile range of indices for postprandial hyperglycemia stratified with SgIo categories.

	Median	IQR	*n*		Median	IQR	*n*	*p*
		SgIo < 2.53				SgIo ≥ 2.53		
1,5-AG, μg/mL	14.8	10.8–23.6	22		21.9	18.6–25.3	28	0.010 *
BGM max, mg/dL	194	178–231	20		183	160–197	25	0.019 *
BGM mean, mg/dL	118	113–124	20		109	104–122	25	0.033 *
BGM SD, mg/dL	28.9	25.5–33.5	20		23.1	21.3–28.8	25	0.005 *
BGM CV, %	25.3	21.4–29.3	20		22.1	18.4–24.2	25	0.012 *
BGM ≥ 200, % ^†^	0.0	0.0–10.7	20		0.0	0.0–0.0	25	0.034 *
BGM ≥ 180, % ^†^	10.2	0.0–17.2	20		5.6	0.0–10	25	0.145
BGM ≥ 140, % ^†^	53.6	27.9–66.7	20		31.6	22.2–41.4	25	0.082
CGM max, mg/dL	206	180–236	19		182.5	164.3–207	24	0.021 *
CGM mean, mg/dL	114	109–119	19		112	104–119	24	0.448
CGM SD, mg/dL	23.1	20.8–25.9	19		17.9	15.6–21.3	24	0.001 *
CGM CV, %	20.2	18.5–23.1	19		16.6	13.9–18.2	24	0.001 *
time CGM ≥ 200, % ^‡^	4.8	0.0–10.0	19		0.0	0.0–4.3	24	0.035 *
time CGM ≥ 180, % ^‡^	15	4.8–36.4	19		4.6	0.0–17.6	24	0.031 *
time CGM ≥ 140, % ^‡^	62.5	42.9–90.9	19		48.6	22.9–77.0	24	0.240
% of ≥200 peaks ^§^	4.8	0.0–10.0	19		0.0	0.0–4.3	24	0.023 *
% of ≥180 peaks ^§^	15.0	4.8–36.4	19		4.6	0.0–17.6	24	0.045 *
% of ≥140 peaks ^§^	62.5	42.9–90.9	19		48.6	22.9–77.0	24	0.183

SgIo, glucose effectiveness determined from 75 g oral glucose tolerance test data; 1,5-AG, 1,5-anhydroglucitol; BGM, blood glucose self-monitoring; SD, standard deviation; CV, coefficient of variation; CGM, continuous glucose monitoring; IQR, interquartile range. ^†^, The percentages of meals with postprandial BGM peaks above the indicated thresholds were shown; ^‡^, The percentages of time when CGM sensor readings were above indicated thresholds were shown; ^§^, The percentages of meals with postprandial CGM peaks above the indicated thresholds were shown; * *p* < 0.05 between SgIo categories.

**Table 5:** The median and interquartile range of indices for postprandial hyperglycemia stratified with DIo levels.

	Median	IQR	*n*		Median	IQR	*n*	*p*
		DIo < 2.06				DIo ≥ 2.06		
1,5-AG, μg/mL	17.6	12.7–23.6	14		21.2	16.3–24.3	36	0.122
BGM ≥ 200, % ^†^	2.6	0.0–9.5	14		0.0	0.0–0.0	34	0.036 *
BGM ≥ 180, % ^†^	8.3	0.0–12.3	14		5.6	0.0–12.2	34	0.475
BGM ≥ 140, % ^†^	36.9	26.4–64.6	14		35.5	22.2–63.1	34	0.725
time CGM ≥ 200, % ^‡^	0.3	0.0–1.4	14		0.0	0.0–0.6	33	0.047 *
time CGM ≥ 180, % ^‡^	1.7	0.3–3.4	14		0.2	0.0–2.3	33	0.040 *
time CGM ≥ 140, % ^‡^	11.2	5.7–17.2	14		8.8	1.9–15.3	33	0.193
% of ≥200 peaks ^§^	4.8	0.0–14.1	13		0.0	0.0–5.8	30	0.162
% of ≥180 peaks ^§^	14.6	3.1–25.8	14		4.8	0.0–19.5	33	0.087
% of ≥140 peaks ^§^	61.9	42.9–80.0	13		56.2	23.3–77.5	30	0.361

IQR, interquartile range; DIo, oral disposition index; 1,5-AG, 1,5-anhydroglucitol; BGM, blood glucose self-monitoring; CGM, continuous glucose monitoring. ^†^, The percentages of meals with postprandial BGM peaks above the indicated thresholds (i.e., 200, 180, and 140 mg/dL); ^‡^, The percentages of time when CGM sensor readings were above the indicated thresholds (i.e., 200, 180, and 140 mg/dL); ^§^, The percentages of meals with postprandial CGM peaks above the indicated thresholds (i.e., 200, 180, and 140 mg/dL); * *p* < 0.05 between DIo categories.

**Table 6:** Proportions of the 1,5-AG < 14 μg/mL category stratified with the SgIo and DIo categories.

Category ^†^			
	SgIo < 2.53	SgIo ≥ 2.53	*p* (SgIo categories) ^‡^
DIo < 2.06	66.7%	0%	0.031 *
DIo ≥ 2.06	30.8%	4.4%	0.047 *
*p* (DIo categories) ^§^	0.192	1.0	

1,5-AG, 1,5-anhydroglucitol; SgIo, glucose effectiveness determined from 75 g oral glucose tolerance test data; DIo, disposition index determined from 75 g oral glucose tolerance test data. ^†^, The subjects were divided by respective cutoffs of SgIo or DIo for detecting the 1,5-AG < 14 μg/mL category (i.e., 2.53 for SgIo and 2.06 for DIo). The proportions of subjects in each group to the whole were shown; ^‡^, Fisher’s exact test is used to compare the proportions of the 1,5-AG < 14 μg/mL category between the SgIo < 2.53 and SgIo ≥ 2.53 categories according to each DIo category; ^§^, Fisher’s exact test is used to compare the proportions of the 1,5-AG < 14 μg/mL category between the DIo < 2.06 and DIo ≥ 2.06 categories according to each SgIo category; * *p* < 0.05.

**Table 7:** Proportions of the SgIo quartile category stratified with the walking (≥8000 steps) day or frequency of noodle ingestion categories.

	SgIo Quartile Category				
Category ^†^	Q1	Q2	Q3	Q4	*p* for Trend ^‡^
					
Walking					0.008 *
<60% of days	29.7%	29.7%	24.3%	16.2%	
≥60% of days	8.3%	16.7%	25.0%	50.0%	
					
Noodle ingestion					0.038 *
<20% of meals	18.5%	18.5%	37.0%	25.9%	
≥20% of meals	40.0%	33.3%	6.7%	20.0%	

SgIo, glucose effectiveness determined from 75 g oral glucose tolerance test data. ^†^, The proportions of subjects in the SgIo quartile categories according to the walking or noodle ingestion category were shown; ^‡^, The Cochran–Armitage trend tests were used if tendencies in proportions of the SgIo quartile categories significantly differ across the walking day category or the noodle ingestion category; * *p* < 0.05.

## Data Availability

Not applicable.
